# Barriers and enablers to screening and diagnosing depression and diabetes distress in people with type 2 diabetes mellitus; protocol of a qualitative evidence synthesis

**DOI:** 10.12688/hrbopenres.12947.3

**Published:** 2020-09-30

**Authors:** Niamh McGrath, Sheena McHugh, Patricia M. Kearney, Elaine Toomey

**Affiliations:** 1 School of Public health, University College Cork, Cork, Ireland; 2 School of Psychology, National University of Ireland Galway, Galway, Ireland; 3 School of Allied Health, University of Limerick, Limerick, Ireland

**Keywords:** Systematic review, Qualitative evidence synthesis, Qualitative research, Depression, Diabetes distress, Diabetes, Screening, Primary care, Guideline adherence

## Abstract

**Background: **Depression and diabetes distress are common in people with type 2 diabetes (T2DM). These conditions are independently associated with poorer T2DM outcomes and increased healthcare utilisation and costs. Questions remain regarding the most appropriate ways of initially detecting depression and diabetes distress in this group. Diabetes guidelines recommend depression screening in primary care for people with T2DM but their implementation in practice is suboptimal. As health care professionals influence detection practices, their perceptions and experiences of these guidelines can improve understanding of aspects of the guidelines that work, and those which are more difficult to implement in practice. This study describes the protocol for a qualitative evidence synthesis of primary care health professionals’ perceived barriers and enablers to screen for and diagnose depression and diabetes distress in people with T2DM.

**Methods and analysis:** Primary qualitative studies will be identified using a systematic search of electronic databases and supplementary searching. We selected ‘best-fit framework synthesis’ as the approach to synthesise primary data using the RETREAT (Review question-Epistemology-Time/Timescale-Resources-Expertise-Audience and purpose-Type of Data) framework. Quality appraisal of primary studies and confidence in the overall review findings will be determined using the CASP (Critical Appraisal Skills Programme) and the GRADE-CERQual (Grading of Recommendations Assessment, Development, and Evaluation Confidence in the Evidence from Reviews of Qualitative research), respectively.

**Discussion: **The planned review will provide the first, single point of reference of the available synthesised qualitative evidence on this topic. It will apply recommended approaches to ensure rigor and robustness of study and contribute meaningfully to understanding of how depression and diabetes distress can be initially detected in people with T2DM. This protocol is registered with the International Prospective Register of Systematic Reviews (PROSPERO) [registration number: CRD42019145483].

## Introduction

“Depression and diabetes distress have both been associated with adverse outcomes among people with type 2 diabetes” ^1– 5^, as well as increased costs to health systems ^6– 8^. Diabetes distress refers specifically to diabetes-related concerns about self-management, perceptions of support, emotional burden, and access to quality health care ^9, 10^ whereas depression is an affective disorder characterised by depressed mood and anhedonia ^11, 12^. Both are highly prevalent in the T2DM population; diabetes distress affects approximately 36% of people with T2DM at any one time ^13^ and depression is estimated as being twice as prevalent in people with T2DM as in the general population ^14^. Yet, depression is substantially undiagnosed in people with T2DM ^15, 16^. For example, national survey data from the United States (US) indicates that of people with type 1 and type 2 diabetes and depression, 45% had never received a depression diagnosis from a general physician ^15^. Less is known about the prevalence of undiagnosed diabetes distress in the T2DM population. However, analysis of observational data of 112 outpatients with diabetes identified that symptoms frequently went unrecorded and health professionals often failed to detect depression and diabetes distress ^16^.

National and international diabetes guidelines recommend routine use of clinical questioning or validated depression and diabetes distress screening tools for initial detection of depression and diabetes distress among people with T2DM in primary care settings ^17– 20^. Although depression and diabetes distress screening is an effective way to accurately identify symptoms in people with T2DM compared to no screening strategy ^21– 23^, implementation of screening guidelines in routine T2DM care is challenging ^24– 27^. In the UK, GPs receive financial reimbursement to administer a depression screening protocol to people with at least one chronic condition, including T2DM ^28^. However, in primary care patients with at least one chronic condition (coronary heart disease, diabetes and previous stroke) in Scotland, depression screening was administered to less than one third (31%) of patients ^24^. In a primary care practice in England, only 72% of diabetes patients received the depression screening protocol, and, less than half of those identified as having possible depression were administered the full symptom measure ^26^.

Implementation of depression screening guidelines in routine T2DM care may be influenced by a lack of consensus around how and when to screen. For example, there is discrepancy around the specific psychosocial difficulties that should be screened for, when screening should be administered and how screening should be administered ^12– 15^ ( *Extended data:* Supplementary File 1). Implementation may also be influenced by patient ^29^ and health care professional specific factors ^27, 30– 32^. Primary care health professionals report common barriers and enablers to screening and diagnosing depression in T2DM as in general populations; mental health stigma, time constraints ^27, 30– 32^, patient-clinician relationship ^30– 32^, and as people with T2DM; normalising depressive symptoms as part of chronic disease ^29, 31, 33^, symptom overlap, and mental health stigma ^29, 31– 33^. Previous studies have also identified that primary care health professionals experience unique barriers and enablers to screening and diagnosing depression and diabetes distress in the T2DM population. These include; perceptions of their role and responsibilities ^31, 32^, the perceived value of screening or clinical questioning in the T2DM population ^31^, and integrating screening protocols into T2DM review visits ^27^.

Qualitative evidence syntheses of patient and health professional factors consolidate findings from multiple primary studies carried out in different contexts in order to; (1) identify the full spectrum of factors which support and hamper guideline implementation, and (2) highlight gaps in knowledge, and areas of saturation where no further primary research is required ^34^. The perspectives of people with T2DM regarding their experiences of depression screening and diagnoses have previously been synthesised ^29, 33^, enabling identification of patient factors influencing detection and diagnosis. While understanding these views is crucial, health professionals are the primary implementers of T2DM depression screening and diagnosis guidelines. Therefore, an in-depth overview of the existing qualitative evidence that captures the perspectives of those responsible for screening and diagnosing depression among people with T2DM is also of paramount importance. Although a previous qualitative evidence synthesis explored general physicians’ perceived barriers and enablers to diagnosing depression in primary care in general ^30^, this has not been previously explored specifically in relation to a T2DM population. Therefore, a qualitative evidence synthesis of the primary care health professional barriers and enablers to screening and diagnosing depression and diabetes distress in people with T2DM can address an important gap in the T2DM literature.

## Protocol

This review will synthesise the available qualitative evidence in the literature that explores primary care health professionals’ views and experiences of screening and diagnosing depression and diabetes distress in people with T2DM.

### Eligibility criteria

***Setting.*** Studies conducted in primary care settings and outpatient diabetes settings, in any country, will be eligible for inclusion.

***Perspective.*** Eligible perspectives are those of any health care professional(s) who screen and diagnose people with T2DM in a primary care setting or in a diabetes outpatient setting. This may include GPs, Practice Nurses, Diabetes Nurse Specialists and Psychologists. Studies including the patient and health professional perspective will be included if the health care professional perspective can be extracted separately from the patient results. Studies that only present the perspectives of people with T2DM or their families will be excluded.

***Phenomenon of interest.*** The phenomenon of interest is the process of screening and/or diagnosing depression and/or diabetes distress in people with T2DM. Studies only focused on the management of people with T2DM and depression and/or diabetes distress will be excluded. Studies about screening, diagnosing and managing people with T2DM and depression and/or diabetes distress will only be included if findings related to screening and/or diagnosis can be extracted separately from results related to management. Studies relating to type 1 and/or type 2 diabetes will be included if studies otherwise meet the inclusion criteria. This includes studies relating to “diabetes” where diabetes type is not specified, mixed sample studies where type 1 and type 2 diabetes are reported or type 1 diabetes only studies. Studies of “chronic conditions” or “multimorbidity” will only be included if findings relating to “diabetes” (not specified or mixed samples), “type 1 diabetes” or “type 2 diabetes” can be distinguished from other conditions included in the study.

***Comparison.*** If results are reported by different types of health professionals, we will compare health professional perspectives.

***Evaluation.*** We will use qualitative evidence to better understand the strategies used by primary care health professionals to screen and diagnose depression and diabetes distress in people with T2DM and to identify primary care health care professional barriers and enablers associated with screening diagnosing depression and diabetes distress in this population.

***Studies.*** Primary research studies that employ qualitative or mixed-methods will be eligible for inclusion. Studies must have used qualitative data collection (e.g. semi-structured interviews, observation) and analyses methods (e.g. thematic analysis, grounded theory). Studies will be peer reviewed journal articles or non-peer reviewed items including unpublished research articles and theses. Non-English language studies, literature reviews and quantitative research studies will not be eligible for inclusion. Multiple-method and mixed-method studies will only be included if qualitative results can be extracted separately from quantitative results. Where the full text is unavailable, we will contact authors in an effort to obtain the full text. All reasonable effort will be made to secure potentially relevant studies, including via interlibrary loans or purchase of relevant studies if required. If it is not possible to obtain full texts, these studies will be excluded.

### Systematic identification of primary research studies

***Search strategy.*** This review will use a combination of systematic searching of the literature using electronic databases and supplementary searching. The following databases will be searched: CINAHL, EMBASE, MEDLINE, PsycINFO, Academic Search Complete, Scopus. These databases and the search terms were selected in consultation with an expert librarian to source peer-reviewed articles across medicine, nursing, gerontology, health services research and psychology disciplines and to identify studies focusing on health professionals’ accounts of screening and/or diagnosing depression and/or diabetes distress in people with T2DM. The search will be conducted in all databases in one day by the lead author. The search strategy for MEDLINE is shown in *Extended data:* Supplementary File 2. Certain search terms are truncated, for example depress* or recogni*, to ensure all spellings are captured. Terms will be adapted for individual databases as needed, for example, MeSH terms will be used for MEDLINE. The use of title and abstract will depend on the individual databases. There will be no restrictions on the years searched in order to retrieve relevant studies from the earliest date possible. This is because despite potential changes in mental health care and perceptions in primary care, individual level factors influencing detection in this population may persist. Persisting factors may include health care professionals’ skills relevant to identification of these problems in the T2DM cohort and health care professionals’ perceptions of patient factors influencing identification may persist despite organisational and systemic changes.

Within QES, the approach to searching should be informed by the overarching aim of the synthesis and the approach to analysis ^35^. We initially selected an “exhaustive” as opposed to a purposive approach to synthesis fitting with our preliminarily selected best fit framework analysis (see data synthesis for details) ^35^. Supplementary searching will be informed by the CLUSTER (Citations, Lead authors, Unpublished materials, Scholar searches, Theories, Early examples, Related projects) approach ^36^. The approach employs techniques relevant to different types of systematic reviews in a systematic manner and offers a systematic approach to supplementary searching ^36^.

***Study screening.*** All references will be imported into Endnote and duplicates removed. The lead author (N.M.G) will screen all titles and abstracts independently using Rayyan QCRI software ^37^. Two reviewers will screen 50% of titles and abstracts each, against the eligibility criteria. When there is no abstract, or it is not possible to determine whether to include an article or not, the full text of the article will be retrieved. The lead author will screen all full-text articles, and two other reviewers will each independently screen 50% of the full text articles against the eligibility criteria. Disagreement between reviewers will be discussed among the reviewers to achieve consensus. If necessary, we will consult with the broader review team until consensus is reached. Results of searching, screening and included studies will be reported using the PRISMA flowchart ^38^.

***Data extraction.*** Data will be extracted using a standardised data extraction form by the lead reviewer. Extracted data for each study will include: the first author, publication year, journal, participant group (type of health professional), setting (country, rural/urban, type of health facility), research methods (method of data collection and analysis, framework used) and outcomes (reported barriers and enablers and related themes). Data will include verbatim quotes from participants and findings reported by the study authors in the results/findings section of included studies ^39^ because best-fit framework synthesis allows for the integration of primary and secondary data ^40^. We will pilot the data extraction form on at least three studies identified from the list of included studies. The lead author will extract data from included papers and two other reviewers will each independently crosscheck 50% of extracted data for consistency and accuracy to minimize potential bias during extraction. Full text articles and extracted data will be imported and managed within QSR NVivo 10 for data synthesis.

### Assessment of quality of included studies

Quality of included studies will be assessed using the Critical Appraisal Skills Programme (CASP) for qualitative research ^41, 42^. In line with the current default position in the conduct of aggregative type QES, assessment of study quality will not be a criteria to exclude studies that otherwise met the inclusion criteria, but used to provide insights into the methods used for data collection and analysis ^43, 44^.

### Data synthesis

The RETREAT (Review question–Epistemology–Time/Timescale–Resources–Expertise–Audience and purpose–Type of Data) framework ^45^ was used to initially select the most appropriate analytical approach. The RETREAT Framework was developed in response to the rapidly growing number of approaches to undertaking qualitative evidence synthesis and to support researchers in selecting appropriate approaches to synthesis. Following initial completion of the RETREAT framework ( *Extended data:* Supplementary File 3), we plan to undertake a ‘best-fit framework synthesis’ ^46^. Best-fit framework synthesis was selected because it offers a pragmatic way to develop intervention theory, is a relatively time efficient method and is suited to an aggregative, as opposed to an interpretative approach to analysis ^46, 47^. However, as the number of studies identified and the heterogeneity of data within identified studies can influence the most appropriate analytic method, we will revisit the RETREAT framework once eligible studies are identified and ensure that best-fit framework synthesis is still appropriate ^48^.

The data synthesis process will be conducted within QSR NVivo 10 to ensure transparency and clarity in the synthesis process ^49^. The lead author will conduct all stages of synthesis from initial coding to interpretation. Two other reviewers will independently analyse a random sample of the data at each stage of the analytic process to enhance consistency of the coding framework and the logic of interpretations. All members of the review team will review and discuss each stage of the synthesis. This will facilitate consensus on the review findings using an iterative approach.

### Confidence in the findings

Confidence in the overall review findings will be determined using the GRADE-CERQual approach given its application to support decision making based on qualitative evidence ^50^ and the availability of resources to support its use ^48, 51– 54^. Application of the CASP forms the methodological limitations component of the GRADE-CERQual assessment. GRADE-CERQual also assesses the relevance of individual review findings (i.e. the extent to which the evidence from the primary studies is applicable to the review question), the coherence of individual review findings (i.e. how well patterns reported are grounded in data from the included primary studies), and the adequacy of the overall review findings (i.e. the richness and quantity of data supporting a review finding). In this way, application of the GRADE CERQual enables the reviewers to understand the influence of aspects of our review such as inclusion of both published and grey literature (methodological component), time of publication, and the population of interest (relevance component) on our overall confidence in individual review findings in relation to the review question. The lead author will carry out each step of the GRADE-CERQual process. Two other reviewers will check for relevance, coherence and adequacy of individual review findings from a selection of data (e.g. from one primary study each). The three reviewers will discuss each phase of the GRADE-CERQual process, and any disagreements will be resolved through discussion and consensus.

### Dissemination of findings

Findings will be submitted to a peer-reviewed journal for publication. The findings will also be used to inform the design of an intervention to support screening and diagnosis of depression and diabetes distress symptoms in people with T2DM attending primary care in Ireland. The findings will be shared with identified stakeholders and at academic conferences.

### Study status

Database searching for primary studies has been completed.

## Discussion

This article describes the protocol of a systematic review to synthesise the available qualitative evidence on primary care health professionals’ views and experiences of screening and diagnosing depression and diabetes distress in people with T2DM. The final review results will provide a single point of reference, which can be utilised by key stakeholders in different ways. For instance, the findings may inform; (1) clinicians on ways to adopt or adapt depression and diabetes distress screening practices, and (2) researchers in the design of evidence-informed healthcare interventions to improve processes for detection and diagnosis of depression and diabetes distress in this population ^34, 55^.

The planned review has a number of strengths and limitations. We will apply recommended approaches to ensure rigor and robustness of the study. Specifically, we will apply the GRADE-CERQual approach to appraise the quality of included studies and enhance the usability of the overall findings, and we have applied the RETREAT framework to select the most appropriate approach to synthesis. However, review findings will be limited by what is reported in the included primary studies as we will not seek original data from the primary studies included. The planned review will not capture challenges associated with screening and diagnosing other pertinent psychological difficulties experienced by people with T2DM (e.g. disordered eating, dementia) ^56, 57^.

Diagnosing depression in primary care populations is challenging in general ^30^. Supporting primary care health professions to detect depression and diabetes distress in people with T2DM is an important step to help address the high prevalence of depression and diabetes distress in this population ^13, 14^. This review will identify those aspects of the available best practice guidelines that work, and those which are more difficult to implement in practice. Ultimately, the findings will improve understanding of how depression and diabetes distress can be appropriately identified in people with T2DM in primary care settings ^34^.

### Reporting guidelines

This paper is reported in accordance with the Preferred Reporting Items for Systematic Reviews and Meta-Analyses-Protocol (PRISMA-P) ^58^. The review will be reported in accordance with the Preferred Reporting Items for Systematic Reviews and Meta-Analyses (PRISMA) ^59^ and the Enhancing Transparency in Reporting the Synthesis of Qualitative (ENTREQ) Research guidelines for systematic reviews ^60^.

We submitted our record to the International Prospective Register of Systematic Reviews (PROSPERO) on August 9th 2019 [PROSPERO ID number 145483]. Due to significant and unexpected demand for the PROSPERO service, our record was processed and registered on November 11th 2019 [PROSPERO registration number: CRD42019145483].

## Data availability

### Underlying data

No data is associated with this article.

### Extended data

Open Science Framework: Barriers and enablers to screening and diagnosing depression and diabetes distress in people with type 2 diabetes mellitus; protocol of a qualitative evidence synthesis, https://doi.org/10.17605/OSF.IO/VF3H2 ^61^.

This project contains the following extended data:

- Supplementary File 1. Summary of depression and diabetes distress screening guidelines for adults with T2DM.
- Supplementary File 2. Search strategy for Medline.
- Supplementary File 3. Use of the RETREAT framework to inform selection of the best-fit framework approach to synthesis.

### Reporting guidelines

Open Science Framework: PRISMA-P checklist for Barriers and enablers to screening and diagnosing depression and diabetes distress in people with type 2 diabetes mellitus; protocol of a qualitative evidence synthesis, https://doi.org/10.17605/OSF.IO/VF3H2 ^61^.

Data are available under the terms of the Creative Commons Zero "No rights reserved" data waiver (CC0 1.0 Public domain dedication).
