# Supplementary material for: Barriers and enablers to screening and diagnosing depression and diabetes distress in people with type 2 diabetes mellitus; protocol of a qualitative evidence synthesis
Source: HRB Open Res. 2020 Sep 30;2:26. Originally published 2019 Oct 25. [Version 3] doi: 10.12688/hrbopenres.12947.3 (PMC7539074; doi:10.12688/hrbopenres.12947.3)
Supplement: Peer review history

# Peer review: Reviewer response for version 3

**DOI:** 10.21956/hrbopenres.14271.r28042

**Published:** 2020-10-06

**Authors:** Andreas Schmitt

**Affiliations:** 1 Diabetes Center Mergentheim, Research Institute of the Diabetes Academy, Bad Mergentheim, Germany

I thank the authors for being responsive to my comments. The article has been revised following the reviewers' suggestions. I have no further comments. Thank you.

Is the study design appropriate for the research question?

Yes

Is the rationale for, and objectives of, the study clearly described?

Yes

Are sufficient details of the methods provided to allow replication by others?

Yes

Are the datasets clearly presented in a useable and accessible format?

Yes

Reviewer Expertise:

Behavioural diabetes research.

I confirm that I have read this submission and believe that I have an appropriate level of expertise to confirm that it is of an acceptable scientific standard.

# Peer review: Reviewer response for version 2

**DOI:** 10.21956/hrbopenres.14082.r27291

**Published:** 2020-04-20

**Authors:** Andreas Schmitt

**Affiliations:** 1 Diabetes Center Mergentheim, Research Institute of the Diabetes Academy, Bad Mergentheim, Germany

Reviewer’s report: The article describes the protocol of a qualitative evidence synthesis regarding barriers and facilitators to screening and diagnosing depression and/or diabetes distress (DD) in T2DM according to HCPs’ reports. The article is well structured and written, the purpose of the research is reasonable and the planned procedures are valid and follow current guidelines.

 Major revision points:

1. The authors underline the need for screening depression/DD in diabetes with evidence suggesting worse health outcomes in affected persons. However, the cited evidence (1–5) does not go beyond cross-sectional associations i.e. does not support depression or DD as predictors of adverse health outcomes. Instead, associations such as depression and HbA1c (likely a bidirectional relationship) or depression and diabetes complications (where depression might be considered to be the consequence rather than antecedence of complications) are referenced. I consider this a poor evidence base for supporting a need for screening, and the existing evidence base is far more conclusive including prospective studies regarding the outcomes HbA1c, incident complications, mortality etc. I would expect a more up-to-date evidence base using studies supporting mechanistic conclusions.
2. The authors claim that “Depression and diabetes distress are independently associated with adverse outcomes among people with type 2 diabetes” (Introduction, first sentence), but the referenced evidence does not support such statement. In fact, four of five cited studies regard cross-sectional associations between either depression alone or DD alone and health outcomes; thus appraisal of independent associations is not possible. Although one of the studies (3) does indeed include both conditions, the findings do not suggest independent associations for depression and DD with the outcome HbA1c (which is a proxy for longer-term health outcomes such as vascular complications as well as acute metabolic complications), instead only DD was associated with HbA1c. On the other hand, the question whether depression or diabetes distress or both are associated with core outcomes in diabetes is highly relevant, and a number of studies (mostly cross-sectional) have indeed addressed this issue. As there is evidence potentially supporting the authors’ claim, I would suggest they either add relevant findings or discount the statement to “both have been associated with adverse outcomes in independent studies” or similar.
3. The authors' precise focus on T2DM is appreciated, and I am aware of the clearly higher prevalence compared to T1DM. However, depression prevalence is similarly increased in T1DM, and DD is also highly prevalent in this group. Accordingly, I do not see why the authors would need to exclude studies regarding T1DM or mixed samples straightaway, while guidelines recommending psychosocial screening and diagnostic refer to T2DM and T1DM, and HCPs may have to deal with both groups so interview reports regarding screening may refer to both diabetes types. I agree that specific inferences and recommendations regarding T2DM or T1DM or both are required, but I cannot see why you should conduct a different study addressing this issue in those with T1DM if one literature search might spot light into both fields. Furthermore, exclusion of T1DM might result in exclusion of all mixed sample studies where results for these groups were not presented separately, despite potential valuable insights to be gained from the HCPs’ reports. I suggest considering inclusion of T1DM-related studies. Please also note that the available evidence base from which to include for this systematic review may be limited (Schumann *et al*.’s study of physicians’ experiences with depression detection in primary care included only 13 studies, so I assume the evidence base specifically for diabetes may be small), thus inclusion of all studies regarding diabetes rather than T2DM only appears reasonable.
4. The literature is searched without restrictions on the year of publication. However, health care interventions, perspectives, reimbursement and consideration of mental health in primary care have changed significantly across the last decades. Newer evidence will therefore probably be of higher relevance/greater interest. A rationale for not excluding potentially outdated studies might be useful.
5. The authors plan to include non-peer reviewed items such as unpublished research articles and theses. A rationale for including potential ‘lower quality’ data would be helpful. Did the authors consider weighting such items somehow differently in order to avoid potential bias when comparing their results to more rigorously evaluated peer-review publications?

Minor points:

- Is the number of relevant studies large enough to suggest a systematic review in this field? How many studies of potential interest did the authors identify actually?
- The authors state that “Where the full text is unavailable, we will contact authors in an effort to obtain the full text. If it is not possible to obtain full texts, these studies will be excluded.” I assume unavailability implies that buying or renting the article would not be possible, correct? Otherwise, I would expect the authors purchase the relevant literature to avoid exclusion of potentially relevant evidence.

Is the study design appropriate for the research question?

Yes

Is the rationale for, and objectives of, the study clearly described?

Yes

Are sufficient details of the methods provided to allow replication by others?

Yes

Are the datasets clearly presented in a useable and accessible format?

Yes

Reviewer Expertise:

Behavioural diabetes research.

I confirm that I have read this submission and believe that I have an appropriate level of expertise to confirm that it is of an acceptable scientific standard, however I have significant reservations, as outlined above.

# Response

**Published:** 2020-09-17

**Authors:** Niamh McGrath

**Affiliations:** University College Cork, Ireland

Response to viewer comment 1 (major): Thank you. We have replaced references 1-5 to include papers demonstrating prospective associations between diabetes distress and adverse type 2 diabetes outcomes and depression and adverse type 2 diabetes outcomes: (p.3, Introduction)

1: Nouwen A, Adriaanse MC, van Dam K, Iversen MM, Viechtbauer W, Peyrot M, Caramlau I, Kokoszka A, Kanc K, de Groot M, Nefs G. Longitudinal associations between depression and diabetes complications: a systematic review and meta‐analysis. Diabetic Medicine. 2019 Dec;36(12):1562-72.

2: Naicker K, Johnson JA, Skogen JC, Manuel D, Øverland S, Sivertsen B, Colman I. Type 2 diabetes and comorbid symptoms of depression and anxiety: longitudinal associations with mortality risk. Diabetes care. 2017 Mar 1;40(3):352-8.

3: Hsu HC, Lee YJ, Wang RH. Influencing Pathways to Quality of Life and HbA1c in Patients With Diabetes: A Longitudinal Study That Inform Evidence‐Based Practice. Worldviews on Evidence‐Based Nursing. 2018 Apr;15(2):104-12.

4: Gonzalez JS, Kane NS, Binko DH, Shapira A, Hoogendoorn CJ. Tangled up in blue: unravelling the links between emotional distress and treatment adherence in type 2 diabetes. Diabetes care. 2016 Dec 1;39(12):2182-9.

5: Aikens JE. Prospective associations between emotional distress and poor outcomes in type 2 diabetes. Diabetes care. 2012 Dec 1;35(12):2472-8.

Response to reviewer comment 2 (major): Author response 2: Thank you. As suggested, we have rephrased the first sentence in the introduction (p.3, Introduction) to read “Depression and diabetes distress have both been associated with adverse outcomes among people with type 2 diabetes”. We have also replaced references 1-5 with references demonstrating causal associations between:

(A) depression and onset of microvascular and macrovascular complications: 

Ref 1: Nouwen A, Adriaanse MC, van Dam K, Iversen MM, Viechtbauer W, Peyrot M, Caramlau I, Kokoszka A, Kanc K, de Groot M, Nefs G. Longitudinal associations between depression and diabetes complications: a systematic review and meta‐analysis. Diabetic Medicine. 2019 Dec;36(12):1562-72.

(B) depression among people with type 2 diabetes and all-cause mortality: 

Ref 2: Naicker K, Johnson JA, Skogen JC, Manuel D, Øverland S, Sivertsen B, Colman I. Type 2 diabetes and comorbid symptoms of depression and anxiety: longitudinal associations with mortality risk. Diabetes care. 2017 Mar 1;40(3):352-8..

(C) diabetes distress and diabetes self-management behaviours and glycaemic control:

Ref 3: Hsu HC, Lee YJ, Wang RH. Influencing Pathways to Quality of Life and HbA1c in Patients With Diabetes: A Longitudinal Study That Inform Evidence‐Based Practice. Worldviews on Evidence‐Based Nursing. 2018 Apr;15(2):104-12.

Ref 4: Gonzalez JS, Kane NS, Binko DH, Shapira A, Hoogendoorn CJ. Tangled up in blue: unraveling the links between emotional distress and treatment adherence in type 2 diabetes. Diabetes care. 2016 Dec 1;39(12):2182-9.

5: Aikens JE. Prospective associations between emotional distress and poor outcomes in type 2 diabetes. Diabetes care. 2012 Dec 1;35(12):2472-8.

(D) depression and diabetes self-management behaviours:

Ref 4: Gonzalez JS, Kane NS, Binko DH, Shapira A, Hoogendoorn CJ. Tangled up in blue: unraveling the links between emotional distress and treatment adherence in type 2 diabetes. Diabetes care. 2016 Dec 1;39(12):2182-9.

5: Aikens JE. Prospective associations between emotional distress and poor outcomes in type 2 diabetes. Diabetes care. 2012 Dec 1;35(12):2472-8.

Response to reviewer comment 3 (major): Thank you for highlighting this point. We agree with the reviewer’s suggestion of including “T1DM-related studies”. For the reasons the reviewer indicated; that primary care health professionals interact with both groups, and the potential limited numbers of T2DM only studies, it was not our intention to exclude mixed T1DM and T2DM studies or diabetes not specified studies. We acknowledge that this is not made explicit in the protocol. To clarify this point, we have added the following sentence (p. 4, Phenomenon of interest): “Studies relating to type 1 and/or type 2 diabetes will be included if studies otherwise meet the inclusion criteria. This includes studies relating to “diabetes” where diabetes type is not specified, mixed sample studies where type 1 and type 2 diabetes are reported or type 1 diabetes only studies. Studies of “chronic conditions” or “multimorbidity” will only be included if findings relating to “diabetes” (not specified or mixed samples), “type 1 diabetes” or “type 2 diabetes” can be distinguished from other conditions included in the study.”  

While inclusion of T1DM specific studies may indeed have enabled comparison of barriers and enablers across diabetes type, as the review progress has progressed somewhat since submission of the protocol, preliminary findings indicate that eligible studies have focused on highly prevalent chronic conditions, such as heart disease or a combination of chronic conditions. This suggests it is unlikely that searches of T1DM specific studies would have returned additional eligible search results to enable meaningful insight into both fields.

To clarify that the potential relevance to the review question of studies considering diabetes not specified, mixed diabetes samples or Type 1 Diabetes samples, may be downgraded, we added the sentence (p.5, Confidence in the findings’): “Application of the GRADE CERQual enables the reviewers to understand the influence of aspects of our review such as inclusion of both published and grey literature (methodological component), time of publication, the population of interest (relevance component) on our overall confidence in individual review findings in relation to the review question.” 

Response to reviewer comment 4 (major): Thank you for raising this point. We have made a number of revisions to the manuscript to clarify the rationale for conducting a search without restrictions on the year of publication:

We added the following sentence to Page 4, ‘Search strategy’: “This is because despite potential changes in mental health care and perceptions in primary care, individual level factors influencing detection in this population may persist. Persisting factors may include health care professionals’ skills relevant to identification of these problems in the T2DM cohort and health care professionals’ perceptions of patient factors influencing identification may persist despite organisational and systemic changes.” 

We added the sentence to Page 5, ‘Confidence in the findings’: “Application of the GRADE CERQual enables the reviewers to understand the influence of aspects of our review such as inclusion of both published and grey literature (methodological component), time of publication, the population of interest (relevance component) on our overall confidence in individual review findings in relation to the review question.” 

Response to reviewer comment 5 (major): Thank you. To clarify our rationale for including potentially ‘lower quality’ data, we added the following to the sentence and supporting reference on Page 5, ‘Assessment of quality of included studies’ section, sentence 2: “In line with the current default position in the conduct of aggregative type QES, assessment of study quality will not be a criteria to exclude studies that otherwise met the inclusion criteria but used to provide insights into the methods used for data collection and analysis43,44. 

And Page 5, ‘Confidence in the findings’: “Application of the GRADE CERQual enables the reviewers to understand the influence of aspects of our review such as inclusion of both published and grey literature (methodological component), time of publication, the population of interest (relevance component) on our overall confidence in individual review findings in relation to the review question.” 

Response to reviewer comment 6 (minor): As stated in the introduction “The perspectives of people with T2DM regarding their experiences of depression screening and diagnoses have previously been synthesised29,33, enabling identification of patient factors influencing detection and diagnosis…Although a previous qualitative evidence synthesis explored general physicians’ perceived barriers and enablers to diagnosing depression in primary care in general30, this has not been previously explored specifically in relation to a T2DM population.” As such, there is a rationale to undertake the study. 

Preliminary searches identified 10 potential studies for inclusion and we think this is ‘large enough to suggest a systematic review in this field’. This is because qualitative evidence synthesis seeks to provide richer, more complete and transferrable findings relating to the synthesis topic. While the number of studies and depth of evidence contributing to an individual review finding may differ across study findings, our application of the GRADE CERQual will enable us to account for this when determining our confidence in individual review findings. 

Response to reviewer comment 7 (minor): Thank you for highlighting this. Further explanation regarding resources available and efforts made to source the full text has been provided on Page 4, ‘Studies’ section. This section now reads “Where the full text is unavailable, we will contact authors in an effort to obtain the full text. All reasonable effort will be made to secure potentially relevant studies, including via interlibrary loans or purchase of relevant studies if required. If it is not possible to obtain full texts, these studies will be excluded"

# Peer review: Reviewer response for version 2

**DOI:** 10.21956/hrbopenres.14082.r27223

**Published:** 2020-03-31

**Authors:** Thordis Thomsen

**Affiliations:** 1 Copenhagen University Hospital Rigshospitalet, Copenhagen, Denmark

Thank you for the opportunity to review this protocol. The protocol describes the background for and methodological approach to reviewing qualitative research on barriers and enablers to systematic screening and diagnosis of diabetes distress and depression in people with T2DM. The review is relevant as it can provide important knowledge for clinicians with regard to developing practice in this important field.

 The introduction provides good argument for conducting the review and the objective of the review is clear.

 The methods for searching, inclusion of studies, assessing methodological quality, synthesizing data and evaluating and the degree of confidence to place in findings is clearly described.

 The discussion presents relevant considerations, including potential limitations of the review.

Is the study design appropriate for the research question?

Yes

Is the rationale for, and objectives of, the study clearly described?

Yes

Are sufficient details of the methods provided to allow replication by others?

Yes

Are the datasets clearly presented in a useable and accessible format?

Not applicable

Reviewer Expertise:

Person-centred interventions for people with T2DM, systematic reviews (quantitative & qualitative), anaesthesiology.

I confirm that I have read this submission and believe that I have an appropriate level of expertise to confirm that it is of an acceptable scientific standard.

# Response

**Published:** 2020-09-17

**Authors:** Niamh McGrath

**Affiliations:** University College Cork, Ireland

The authors thank the reviewer for taking the time to review this paper.
